# Opening neural gateways: old dog now has new tricks

**DOI:** 10.3389/fphar.2024.1389383

**Published:** 2024-07-01

**Authors:** Jiamin Peng, Yong Han, Ligang Wang

**Affiliations:** ^1^ Department of Clinical Laboratory, Tongde Hospital of Zhejiang Province, Hangzhou, Zhejiang, China; ^2^ Department of Thoracic Surgery, Zhejiang Provincial People’s Hospital, Affiliated People’s Hospital, Hangzhou Medical College, Hangzhou, China; ^3^ Key Laboratory of Tumor Molecular Diagnosis and Individualized Medicine of Zhejiang Province, Hangzhou, Zhejiang, China; ^4^ Cancer Center, Department of Ultrasound Medicine, Zhejiang Provincial People’s Hospital, Affiliated People’s Hospital, Hangzhou Medical College, Hangzhou, Zhejiang, China

**Keywords:** BBB, pharmacology, BBB imaging, drug, neurology, Alzheimer disease

## Introduction

The blood–brain barrier (BBB) is a physical and biochemical boundary between the parenchyma of the central nervous system (CNS) and the bloodstream (Uchida et al., 2023). The BBB is a complex network of endothelial cells that line the brain’s capillaries, fortified by tight junctions, surrounded by a basement membrane, and supported by pericytes, microglia and astrocytes (Hynynen, 2024). This intricate structure serves as a guardian of the brain’s internal environment, meticulously regulating the passage of substances from the blood to the brain. Its selective permeability is crucial for maintaining neuronal homeostasis and shields the CNS from viruses, bacteria, and neurotoxins (Uchida et al., 2020; Terstappen et al., 2021; Uchida et al., 2022). However, this protective mechanism poses a significant challenge for the delivery of therapeutic agents to the brain. The BBB prevents 98% of small-molecule drugs and 100% of large-molecule drugs from entering the CNS, limiting treatment options for a wide range of neurological conditions (Hynynen, 2024). For instance, neurodegenerative diseases like Alzheimer’s and Parkinson’s require therapeutic agents that can target the brain’s affected regions. Similarly, treating brain tumors demands that chemotherapeutic agents traverse the BBB to reach malignant cells (Partridge et al., 2022).

The challenge of BBB permeabilization has spurred a multitude of research endeavors aimed at developing techniques to safely and transiently open this barrier, facilitating the delivery of drugs to the brain. Of them, non-invasive technologies such as MRI- guided focused ultrasound (MRgFUS) and Tumor Treating Fields (TTFields, based on Electric Field technology) emerge as promising solutions, offering a blend of safety, efficacy, and patient-centric design (Guo et al., 2022; Salvador et al., 2022; Meng et al., 2023).

## Opinion

Old drugs that are previously excluded from the brain could be delivered to CNS through these temporarily BBB opening technologies for the treatment of brain diseases. In the future, new biopharmaceuticals such as antibody drugs will also be developed and delivered to the brain to treat CNS disorders including Alzheimer’s disease, Parkinson’s disease and glioblastomas.

## Evidence to support this opinion

Recently, various technologies have been developed to deliver drugs to the CNS, some of which have entered clinical trials. In previously experimental models, the application of focused ultrasound to open the BBB resulted in a level of five to eight times high of aducanumab delivery to targeted brain regions in comparison to the untreated regions of the brain (Leinenga et al., 2021; Kong et al., 2022).

In an investigator-initiated, prospective, open-label, single-group, single-institution, proof-of-concept trial involving three patients that published in *New England Journal of Medicine*, Rezai et al. utilized magnetic resonance imaging (MRI)–guided focused ultrasound to temporarily open the BBB and enhance the delivery of aducanumab, which is an amyloid-binding monoclonal antibody with limited penetration of the BBB(2). The authors found that, during the 6-month combination-treatment phase, the reduction in the level of Aβ was numerically greater in regions treated with focused ultrasound than aducanumab therapy alone in homologous regions that were not treated with focused ultrasound (Rezai et al., 2024).

The BBB is a major challenge for malignant gliomas treatment, which limits the penetration of most chemotherapeutic drugs. Low-intensity pulsed ultrasound with concomitant administration of intravenous microbubbles (LIPU-MB) could temporarily open the BBB for drug delivery. In a dose-escalation phase 1 clinical trial in adults with recurrent glioblastoma that published in *The Lancet Oncology*, Sonabend et al. reported that the application of LIPU-MB to large areas of the brain to deliver albumin-bound paclitaxel across the BBB is safe and well tolerated. This work provides the first direct evidence that LIPU-MB could enhance the delivery of systemically administered large molecule drugs to the brain and significantly increase its brain concentration in human (Sonabend et al., 2023).

TTFields is a physical therapy that uses low-intensity and moderate frequency and alternating electric fields to inhibit tumors. The US Food and Drug Administration have approved TTFields for treating recurrent or newly diagnosed glioblastoma and malignant pleural mesothelioma (Tanzhu et al., 2022). Besides, TTFields could also reversibly permeabilize the BBB and enhance the delivery of BBB restricted drugs *in vivo* and *in vitro* (Salvador et al., 2022).

In conclusion, by making advantage of BBB opening technologies, old drugs that previously have limited access to the brain could be reconsidered for the management of CNS disorders.

## Discussion and conclusion

The quest to overcome the BBB’s restrictive influence on drug delivery has led to the development of a diverse array of permeabilization techniques, each with its unique strengths and limitations. For instance, MRgFUS offers precise control over the area of the BBB disruption, allowing for targeted treatment with minimal impact on surrounding tissues. The non-invasive nature of this technology aligns with the growing demand for patient-friendly treatment modalities. The integration of MRI allows for real-time monitoring and precise targeting of ultrasound energy, enhancing the safety and efficacy of BBB permeabilization (Uchida et al., 2023). However, MRI is needed because energy from ultrasound could still potentially cause damages to brain cells and nerve. Analogous to LIPU, the impacts of MRgFUS are transient, demanding vigilant oversight to mitigate the risk of ultrasound-induced tissue harm. Moreover, MRgFUS technology entails substantial costs and necessitates operation by specialized personnel within clinical environments.

The continuous application of TTFields can be easily integrated into patients' daily lives, offering a non-invasive and ongoing treatment option. However, the necessity for uninterrupted therapy with TTFields could significantly affect patients' daily lives, posing challenges to their comfort and overall quality of life. Moreover, the enduring consequences of electric field exposure on non-targeted, healthy brain tissue remain to be thoroughly understood, necessitating extensive research. Additionally, the practical aspects of therapy, including the need for patients to shave their heads and secure electrodes to their scalp, may lead to discomfort, skin irritation, or rashes, further complicating the treatment experience.

In summary, large-volume BBB opening technologies are safe and reproducible, which means such procedures could be repeated over multiple cycles of chemotherapy. Benefit from these technologies, old drugs and large size biopharmaceuticals that previously could not pass BBB now can be easily delivered to the brain and could be considered for the treatment of CNS disorders such as Alzheimer’s disease, Parkinson’s disease and glioblastomas. Moreover, the R&D paradigm of drugs for brain diseases would change due to the repaid progress of BBB opening technologies. Nonetheless, since BBB opening or dysfunction might have certain side effects (Uchida et al., 2020; Uchida et al., 2022), the dynamic real-time monitoring of BBB permeability through BBB imaging technologies is highly required to ensure the precise sites of action and length of BBB opening time (Uchida et al., 2023).
